# Chasing long‐range evolutionary couplings in the AlphaFold era

**DOI:** 10.1002/bip.23530

**Published:** 2023-02-08

**Authors:** Theodoros K. Karamanos

**Affiliations:** ^1^ Department of Life Sciences, Faculty of Natural Sciences Imperial College London London UK

**Keywords:** AlphaFold, coevolution, computational biology, intrinsically disordered proteins, protein structure

## Abstract

Coevolution between protein residues is normally interpreted as direct contact. However, the evolutionary record of a protein sequence contains rich information that may include long‐range functional couplings, couplings that report on homo‐oligomeric states or even conformational changes. Due to the complexity of the sequence space and the lack of structural information on various members of a protein family, it has been difficult to effectively mine the additional information encoded in a multiple sequence alignment (MSA). Here, taking advantage of the recent release of the AlphaFold (AF) database we attempt to identify coevolutionary couplings that cannot be explained simply by spatial proximity. We propose a simple computational method that performs direct coupling analysis on a MSA and searches for couplings that are not satisfied in any of the AF models of members of the identified protein family. Application of this method on 2012 protein families suggests that ~12% of the total identified coevolving residue pairs are spatially distant and more likely to be disordered than their contacting counterparts. We expect that this analysis will help improve the quality of coevolutionary distance restraints used for structure determination and will be useful in identifying potentially functional/allosteric cross‐talk between distant residues.

## Graphical abstract

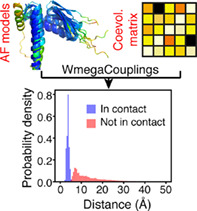

## 1 INTRODUCTION

Since its invention, the field of protein science has seen many breakthroughs. The most recent involves the accurate prediction of protein structure from the primary amino acid sequence alone,^[^ ^1^ ^]^ that lead to the generation of proteome‐wide structural models. Although these models contain important information about the most probable protein state, questions like protein dynamics, conformational changes, protein excited states^[^ ^2^, ^3^, ^4^ ^]^ and protein–protein interactions still pose significant challenges for protein chemists. Here, I will discuss how AlphaFold (AF) models may help formulate hypotheses that could drive experimental studies in order to address some of these issues.

Proteins need to balance structural integrity with plasticity that allows them to respond to environmental stresses, and biological function. Therefore, amino acid covariation within a family of evolutionary related proteins serves to preserve the overall fold, allow potential conformational changes and to maintain protein activity.^[^ ^5^ ^]^ In cases where the probability of substitution in position *i* is influenced by the state of position *j* residues *ij* are considered to coevolve. Such coevolutionary couplings are reflected in multiple sequence alignments (MSAs) and thus, many methods have been developed in order to detect sequence covariation within protein families.^[^ ^6^, ^7^ ^]^ Distinguishing between direct coupling interactions and residues that are coupled indirectly, through chaining effects (such as spurious *ik* couplings in a *i–j–k* interaction) has been a significant challenge which has experienced significant progress in recent years.^[^ ^6^, ^7^, ^8^ ^]^ The success in detecting direct residue–residue coevolutionary couplings is amongst the key driving forces behind the achievements of machine learning methods able to predict the structure of a protein.^[^ ^1^, ^9^, ^10^ ^]^ However, directly coupled residues do not have to be always close in space. Previous work has suggested that coevolution at distal sites could arise from other sources such as: allosteric interactions,^[^ ^11^ ^]^ negative design^[^ ^12^ ^]^ and codon effects.^[^ ^13^ ^]^ More recently it was shown that many directly coupled residues that are not close in space could be explained by structural variation within a family of proteins.^[^ ^25^ ^]^ This means that one would need high resolution structures for many/all family members in order to identify an artefact‐free set of coevolutionary restraints, a task that is almost impossible. In terms of using coevolutionary couplings as distance restraints the problem is manageable since iterative structure calculation protocols are able to easily detect restraints that are consistently violated and exclude them.^[^ ^14^ ^]^ However, detecting real, structurally distant, long‐range couplings that may report on allosteric mechanisms and/or additional protein states remains much harder. Building on the success of AF and the release of AF database I developed a computational approach (termed WmegaCouplings) that performs direct coupling analysis on a given MSA, selects representative AF models of the identified protein family and looks for couplings that cannot be satisfied in any of the selected models. The method was applied to 2012 protein families to reveal that ~12% of the coevolving pairs of residues are not in close contact. Statistical analysis of the distal coevolving pairs hinted that these are more likely to be disordered than their contacting counterparts. Specific examples that report on structural dynamics, homo or hetero‐ oligomerization or potential allosteric regulation are discussed.

## 2 METHODS

### 2.1 Simulations using synthetic sequences

An input sequence of length *L* was created manually and converted to a set of integers corresponding to each amino acid type. The corresponding blosum62‐based substitution matrix consisting of *L* columns by 20 rows was generated in Python. Pairs of coevolving columns and the associated residue types were created manually (see below). In each mutation cycle each element on a column of the substitution matrix was multiplied by a random integer 1 < *m* < 10, and the column element with the maximum value was selected as the mutated residue after back‐encoding. Thus, with no coevolution introduced, this method essentially captures the amino acid conservation encoded in the substitution matrix. To introduce coevolution, a positional and state dependent term was introduced. For instance, if a lysine appears at position 11 then the probability of finding a glutamate at position 21 increases by a coupling constant *c*. After *N* cycles of mutations, the resulting MSA is passed to CCMpred for detection of coevolving residue pairs.

### 2.2 Input dataset

4000 protein sequences that correspond to PFAM families with a representative structure in the PDB have been identified using the SIFTS tool.^[^ ^15^ ^]^ For each family the sequence of the corresponding PDB entry (which is likely lacking flexible regions) was downloaded and used as input for analysis. Sequences with an *N*_eff_ (Equation 1) of below 6 or short sequences below 20 amino acids were excluded during analysis.

### 2.3 Identification of coevolving pairs in single chains

For each query sequence an MSA was constructed using HHblits with parameters: ‐min_prefilter_hits 1000 ‐cpu 4 ‐n 2 ‐e 0.001 ‐p 20 with the UniRef20 as a target database (downloaded in November 2022). The resulting MSA was evaluated in terms of the number of effective sequences (*N*_eff_) as calculated by HHblits^[^ ^16^ ^]^:

(1) $$N_{\mathrm{eff}}=\frac{1}{\sqrt{L}}\sum_{n=1}^{N}\frac{1}{1+\sum_{m=1,m\neq n}^{N}I\left[S_{m,n}\geq 0.8\right]},$$

where *L* is the length of the query sequence, *N* the number of sequences in the MSA, *S*_*m,n*_ is the sequence identity between the sequences *m* and *n*. Sequences with *N*_eff_ < 6 or *L* < 20 where excluded from further analysis. The resulting MSA was passed to CCMpred^[^ ^7^ ^]^ that was run with default parameters and the python script ‘top_couplings.py’ was run to extract the top 20 coevolving pairs (−n 20) that are at least 7 residues apart (−s 7). Note that care has to be taken when one (or both) of the predicted coevolving residues is highly conserved as these positions barely change in the MSA and can give rise to artefacts. The accession numbers of the top 50 hits were extracted from the output of HHblits and the AF database was checked for available models. The average pLDDT score over the atoms corresponding to the query sequence was calculated and the model was discarded if it had an average pLDDT score that was less than 60. The cutoff value of 60 acts as an AF model quality check and was selected here as the dataset includes sequences with corresponding PDB structures, but this value may need to be changed if the query sequences are largely unfolded. Next, distances (*d*) between any non‐hydrogen atoms of the identified coevolving pairs where calculated and the restraint was marked as violated if *d* > 5.0 Å. Restraints that are consistently violated in all 50 AF models are then reported as coevolving but non‐contacting hits. Data were collected on a standard desktop machine with four CPU cores and together with the python‐based method are freely available at https://github.com/karamanoslab/wmegacouplings.

### 2.4 Identification of coevolving pairs in complexes

The two query sequences that potentially form a complex were searched against the Uniref20 database using HHblits with parameters ‐maxfilt 100,000,000, −diff inf, −all. The two resulting individual MSAs were combined into a paired MSA using a phylogeny approach similar to that of Zhou et al.^[^ ^17^ ^]^ Briefly sequences were grouped by species and sorted according to their homology with the input sequence. *N* species‐specific sequences from the first MSA were then concatenated to *N* sequences from the second MSA to form the paired MSA which was then passed to CCMpred. All subsequent analysis was the same as the one performed for single chains with the exception that the distance violation cutoff was set to 7 Å.

## 3 RESULTS AND DISCUSSION

### 3.1 Assessing the ability of direct coupling methods to detect weak couplings

**FIGURE 1:**
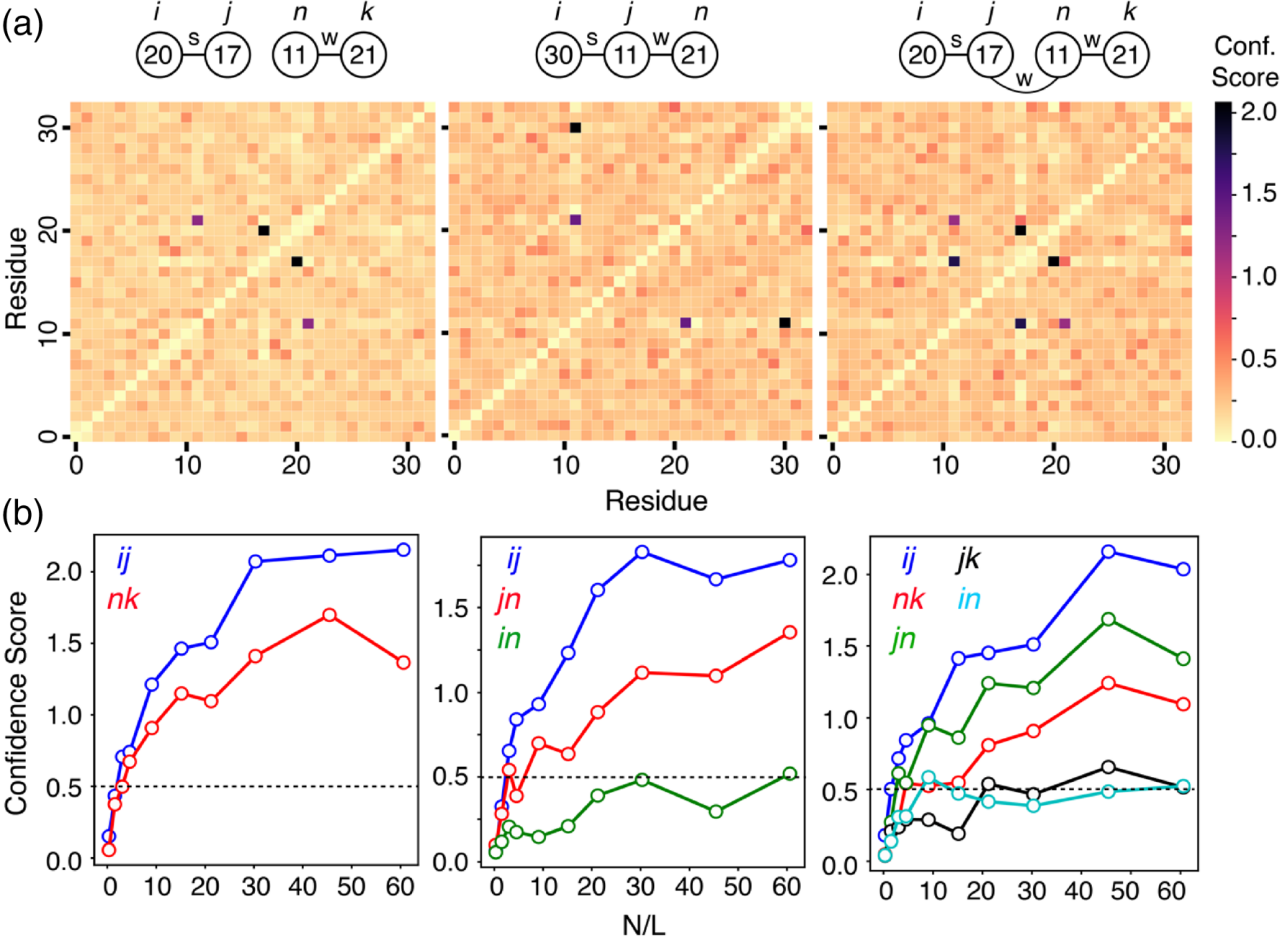
Detecting directly coupled residues using synthetic sequences. (a) CCMpred calculated coevolution matrices plotted as heat maps for the corresponding interaction schemes shown on the top of each panel. Each line corresponds to residues that are strongly (labelled as s, *c* = 10) or weakly (labelled as w, *c* = 4) coupled. The confidence score of the coupling prediction at each position on the heat map (shown as different colours) is plotted against the *N*/*L* ratio in (b). *N*, *L* denote the number of rows, columns in the MSA, respectively. The largest value observed for the background noise in the coevolution matrices is ~0.5, shown as a dotted line in (b).

Long‐range functional or allosteric couplings might be weak (in comparison to couplings between residues that are close in space) or be involved in networks of coupled residues in which each layer may have a different coupling strength. Previous studies have evaluated the performance of these methods and their ability to detect direct from indirect effects in detail.^[^ ^8^, ^18^, ^19^, ^20^ ^]^ In this section, and mainly for didactical reasons, I will carry out straightforward simulations in order to access the ability of current methods to deal with various coevolution scenarios and coupling strengths. For this purpose, synthetic sequences were used to generate simulated MSAs which then acted as input for direct coupling analysis. I focus on CCMpred^[^ ^7^ ^]^ as a coupling detection method since it has emerged as a state‐of‐the art tool with advantages in detection sensitivity and speed. To perform these simulations an initial sequence (>20 residues long) was mutated randomly using a blosum62 substitution matrix. To create coevolving pairs the probability of substitution at position *j* was increased by a coupling constant c_*ij*_ only if a condition for the state of position *i* is satisfied (i.e. if position *i* = 3 is a lysine increase the probability of finding a glutamate at position *j* = 10 by c_*ij*_). The generated sequences/MSAs even though they are inherently much simpler than their naturally occurring counterparts are able to capture the key elements of sequence conservation and coevolution and can be used for analysis by CCMpred. Another advantage of this approach is that altering the number of columns (*L*) and/or the number of rows (*N*) in the simulated MSAs is trivial, making easy to assess the effect of MSA length or depth on the fidelity of coupling detection.^[^ ^21^ ^]^ Using this approach, various coupling interaction schemes and strengths were tested as shown in Figure 1a. In the simplest case of two coupled residue pairs (Figure 1a,b, left‐most panels) CCMpred is able to confidently detect strong (*c*_*ij*_ = 10) and weak (*c*_*ij*_ = 4) interactions as long as the *N/L* ratio is above ~3 consistent with previous reports.^[^ ^7^, ^21^ ^]^ Note that a coupling strength of 10 is comparable to the highest value in the blosum62 substitution matrix (a tryptophan to tryptophan substitution has a score of 11), while a value of 4 is close to the average positive blosum62 value. Interestingly, in a more complicated chain reaction between strongly coupled residues *ij* (residues 30–11) and weakly coupled residues *jn* (residues 11–21) no spurious indirect *in* couplings (residues 30–21) appear even at high *N/L* ratios (>30) (Figure 1a,b middle panels). This is also the case in even more complicated coupling schemes (Figure 1a,b right‐most panels) where only couplings between directly coupled residues (residues 20–17, 11–21, 17–11) but not indirect couplings (residues 20–11, 17–21) can be detected. More ‘real‐life’ parameters such as high and low entropy MSA columns can be easily incorporated in the above analysis but this is beyond the scope of this article.^[^ ^22^ ^]^ Given the sensitivity of CCMpred in detecting directly coupled residues^[^ ^7^ ^]^ we then proceeded in devising a computational method designed to detect couplings between residues that are not in spatial proximity.

### 3.2 Detecting coevolving residues that are distant in structure

**FIGURE 2:**
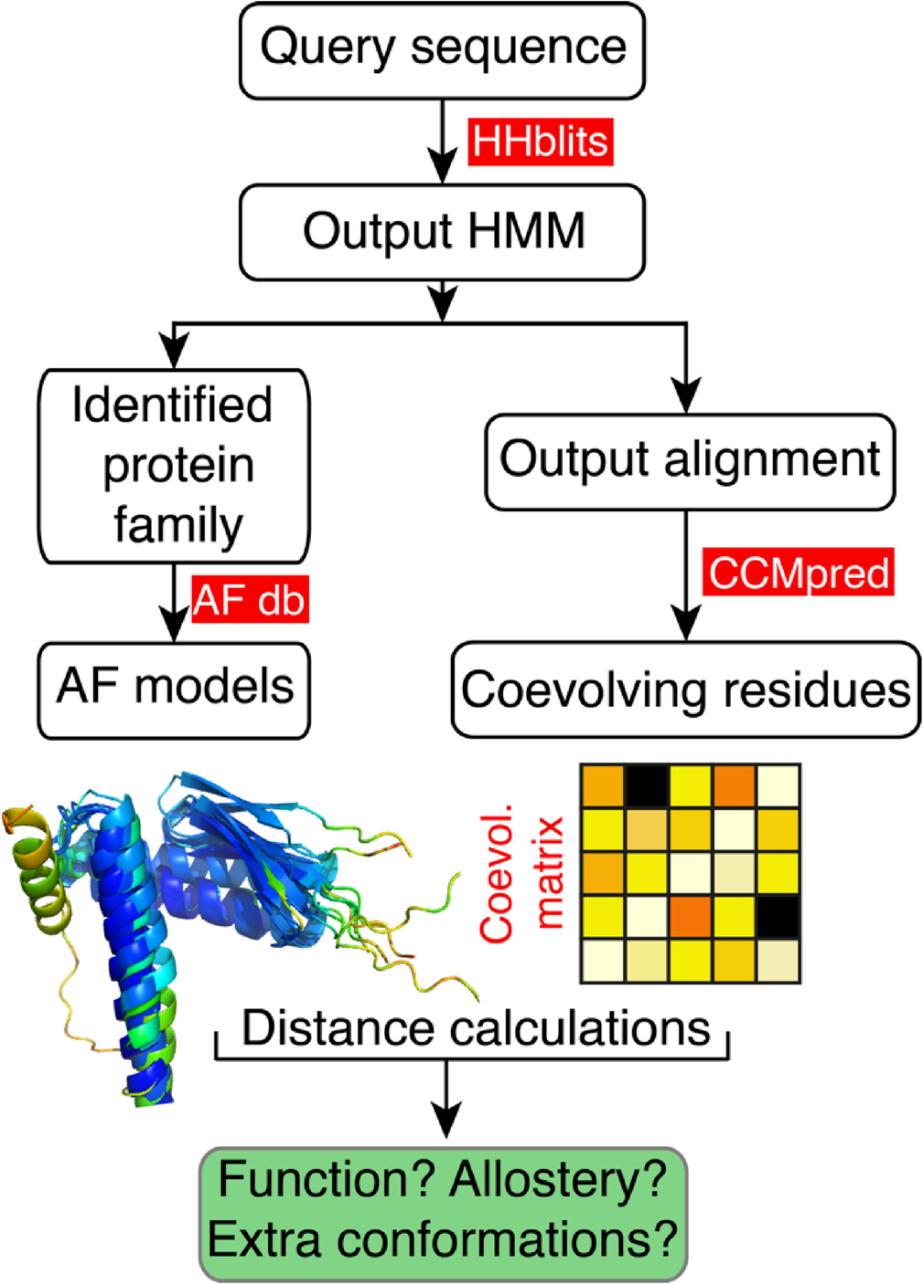
Workflow of the proposed method. Starting from a query sequence (or MSA) HHBlits^[^ ^16^ ^]^ is used to generate an HMM that accurately identifies the protein family that the input sequence belongs to, and outputs an MSA. The AF database is then mined to get AF models of the identified family that have highly similar features as the input sequence and CCMpred^[^ ^7^ ^]^ is used to predict the coevolution matrix from which the highest scoring couplings are extracted. Distances between coevolving residues are then calculated using the selected AF models to identify pairs that are not is spatial proximity and thus could report on protein function, allostery or additional conformational states.

The presented computational approach is built around a protein sequence alignment of a query sequence by using hidden Markov models performed by HHblits^[^ ^16^ ^]^ followed by detection of coupled columns using CCMpred.^[^ ^7^ ^]^ To generate the MSA, HHblits is run against all available sequences in the Uniref100 database using parameters that are very similar to the ones used by AF.^[^ ^23^ ^]^ The resulting MSA is assessed in terms of its diversity (see Methods) and then passed to CCMpred for detection of coevolving residues. The top‐scoring couplings are extracted from the coevolution matrix and the distances of the corresponding residues are calculated in the AF models of the HHblits‐identified homologous proteins. A contact is identified if any two non‐hydrogen atoms are within 5.0 Å of each other. If a coevolving pair of residues are not in contact in any of the AF models they are marked as a hit (Figure 2). To ensure that the generated MSAs are deep enough and thus can be used for reliable prediction of coevolving residues the analysis was restricted to families that have an effective number of MSA sequences (*N*_eff_ as calculated by HHblits) larger than 6. The dataset tested here consisted of sequences belonging to 4000 PFAM^[^ ^24^ ^]^ families of which 2012 pass the MSA diversity criteria. The Python‐based code used to generate the results presented in the following sections is freely available at https://github.com/karamanoslab/wmegacouplings. Typical run times that essentially depend on the speed of HHBlits and CCMpred were 15–20 min for a 150‐residue protein on an average desktop computer with four CPU cores.

From the 2012 protein families analysed using this method, 1304 (~65%) had at least one coevolving pair of residues that are not in spatial proximity. Overall, ~12% (4888 out of 40,240) of the predicted coevolution restraints over all families are not satisfied in any of the AF models. This is in line with previous results showing about 10%–25% violations when using high resolution crystal structures.^[^ ^25^ ^]^ Previous studies have also shown that lower resolution crystal structures tend to exhibit a larger number of coevolving residues that are not in contact.^[^ ^25^ ^]^ Moreover, the frequency of non‐contacting coevolving pairs is larger for families that have a large number of homologue sequences.^[^ ^25^ ^]^ These two observations suggest that inaccuracies in structure determination and structural variation within a given family are detrimental for analyses as the one presented here. Fortunately, the use of AF models alleviates both issues. As accurate‐enough models are available for many homologue sequences in most cases, the method presented here achieves predictions comparable with those obtained from <2 Å resolution crystal structures.^[^ ^25^ ^]^

**FIGURE 3:**
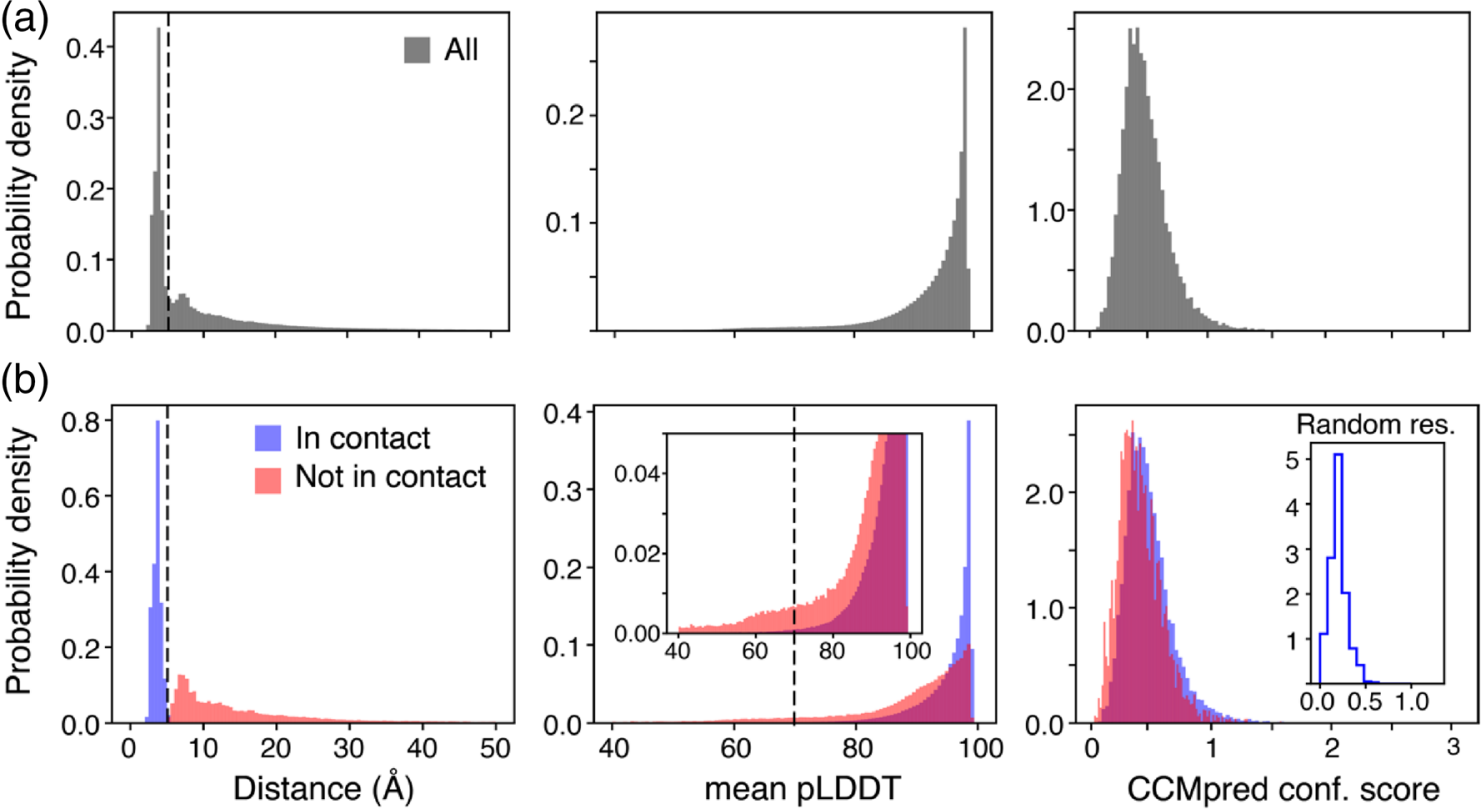
Analysis of coevolving residues. Distance (left), mean pLDDT (middle) and CCMpred score (right) distributions for all coevolving pairs analysed (a, grey) or coevolving residues that are in contact in at least one AF model (b, blue) or not in contact in any of the AF models used in the analysis (b, red). The data have been normalised such that the area under the curve for all three cases equals to unity. The mean pLDDT score refers to the mean pLDDT score between the two closest atoms of the coevolving residue pair. The black dashed line in the left most panels represents 5.0 Å that was used as a contact distance cutoff. The dashed line in the middle panels represents a mean pLDDT score of 70. A zoom‐in focusing on lower pLDDT scores is shown in the middle panel in (b). The inset in the right panel of b represents the distribution of CCMpred scores for randomly selected residues. The dataset of the 2012 PFAM families was used.

The majority of the unsatisfied contacts lie at 6.5–7.5 Å but distances (*d*) can extend up to 20 Å or more (Figure 3a,b left panels). To investigate the origins of these unsatisfied distances it is informative to evaluate the positional precision of the corresponding residues/atoms. Coevolving residues that are in close contact have their positions well determined as judged by the AF expected position error (or pLDDT score) which has a value of 95 ± 6 for these residues (Figure 3b, middle panel). On the other hand, the distribution of pLDDT scores for violated restraints is broader with a mean value of 85 ± 13 (Figure 3b, middle panel). As pLDDT scores can be used as a proxy for protein disorder,^[^ ^26^, ^27^ ^]^ this result could suggest that distal coevolving residues may be involved in alternative protein conformations. Indeed, a small degree of anti‐correlation is observed between *d* and pLDDT which share a Pearson's correlation coefficient of −0.37. To further explore the link between pLDDT and *d*, the distance distributions of residues with different pLDDT scores were evaluated (Figure S1). Pairs of residues with an average pLDDT score of more than 85 showed a distance distribution with a clear maximum ~6.5 Å (Figure S1a), in contrast with residue pairs with an average pLDDT score of less than 70 that exhibit a broad distance distribution that extends up to 50 Å (Figure S1b,c). It is also important to assess whether the quality of CCMpred predictions for contacting and non‐contacting residues is comparable. CCMpred scores of coevolving residues that are not in contact show a normal distribution centred around ~0.41 that is similar to that of contacting coevolving residues (centred around ~0.49). These distributions do not resemble the distribution of CCMpred scores for randomly selected residues (centred around ~0.2) (Figure 3b, left panels) suggesting that the selected coevolutionary couplings are well above the noise of the prediction. In the following section a few examples of identified coevolving but apparently, non‐contacting residues and their possible interpretations will be given.

### 3.3 Non‐contacting coevolving residues: Homo‐oligomerization, dynamics or function?

**FIGURE 4:**
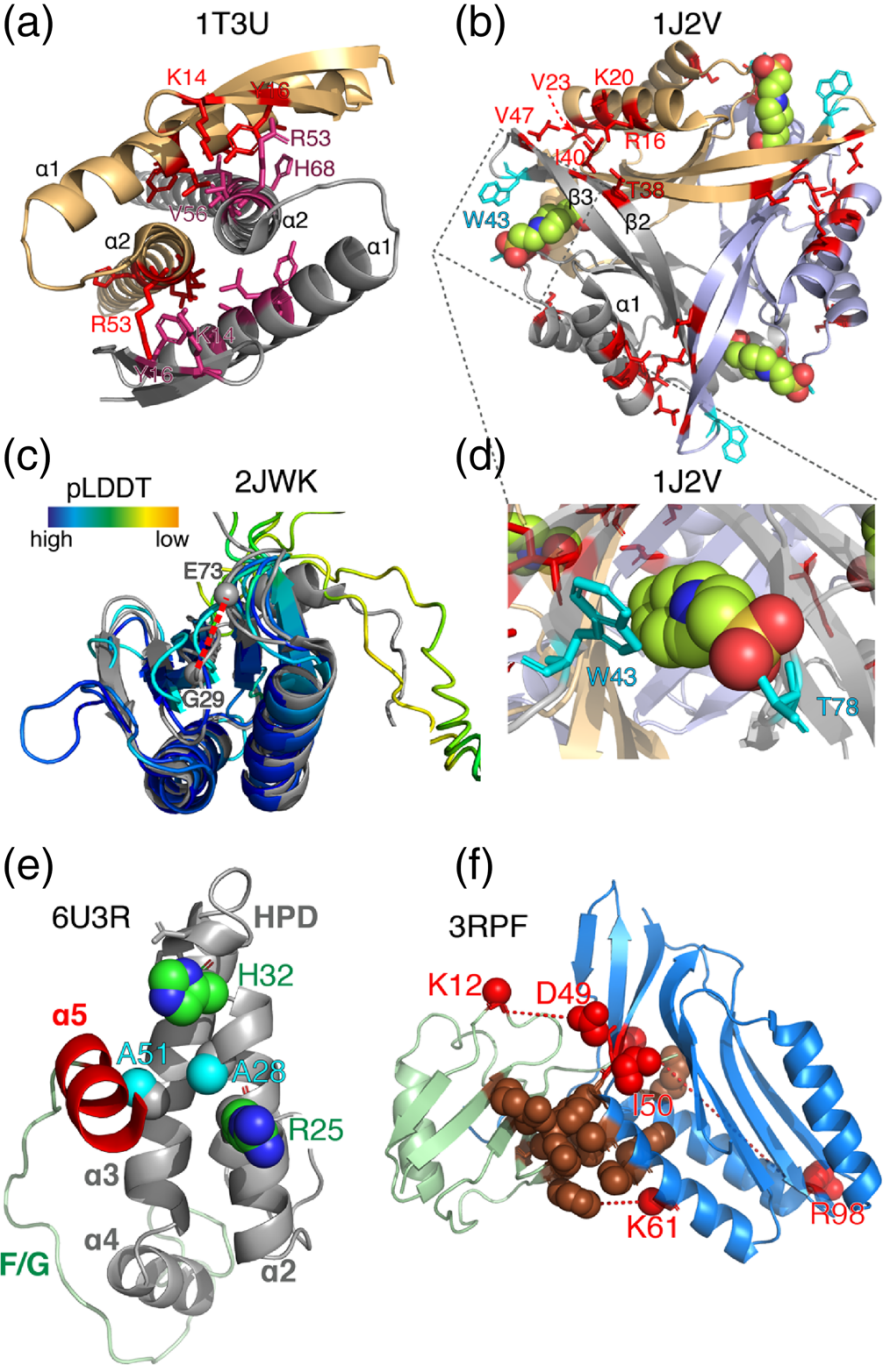
Examples of coevolving residues that are not in contact. (a–b) Structures of homo‐oligomeric proteins ZapA^[^ ^28^ ^]^ (a) and CutA^[^ ^29^ ^]^ (b) with each protomer shown in different colour. Coevolving residues across the oligomeric interface are shown as sticks and highlighted in cyan/blue in (a) and red in (b). (c) Cartoon representation of the periplasmic domain of TorI1. Three AF models (A0A259LVH2, A0A011P9K9, A0A023Y119) are coloured according to their pLDDT scores and two crystal structures (2JWK^[^ ^30^ ^]^ and 5BY4^[^ ^31^ ^]^ are shown in grey. The Cα atoms of coevolving residues glycine 29 and glutamate 73 are highlighted in spheres. (d) Zoom‐in in the ligand binding region of CutA. The ligand is shown in spheres and residues 43 and 78 are shown in cyan sticks. (e) The structure of the J domain of DNAJB6b.^[^ ^32^ ^]^ Helices α1, α2, α3, α4 shown in grey, helix α5 is shown in red and the flexible F/G linker in light green. The two pairs of non‐contacting coevolving residues are shown in green (histidine 32—arginine 25) or in cyan spheres (alanine 51—alanine 28). (f) Structure of the hetero‐complex between subunits 1 and 2 of the molybdopterin converting factor (3RPF). Coevolving residues that lie in the interface between the two subunits are shown as brown spheres and those that are not part of the binding interface are shown in red.

Although many protein families with one or more coevolving pairs of residues that cannot be explained by the corresponding structure have been identified using the above method, in this section I focus on a few examples in order to demonstrate the potential origins of coevolution in these cases. A more thorough analysis of the matter has been performed before,^[^ ^25^ ^]^ although this was limited to available crystal structures at the time, and could not assess structural variability within a protein family as effectively as it is done here. As the functional form of many proteins is a homo‐oligomer, strong coevolution is observed between residues that lie in the oligomeric interfaces. About 35% of the coevolving residues that are distant on the monomeric structure are involved in homo‐oligomerisation.^[^ ^25^ ^]^ Indeed, our dataset contains many such examples as shown in Figure 4a,b. The bacterial cell division protein ZapA (1TU3 or 1W2E)^[^ ^28^ ^]^ and the protein CutA (1J2V)^[^ ^29^ ^]^ function as a tetramer/trimer, respectively, and coevolution has ensured that these oligomeric states are maintained. CutA has 5 out of 20 coevolution restraints that do not fit the structure of the monomer. Four of these residues locate in the β2, β3 strands that coevolve strongly with residues in helix α1 as these two parts of the protein form the trimer interfaces. For ZapA, seven coevolving pairs of residues are found in the interfaces between the monomers. The distances between those two sets of residues are quite large in the monomeric subunits, giving rise to more than 15 Å distance violations in Figure 3b. Notably, the pLDDT scores for all of these residues in both CutA and ZapA AF models are >85.

Although AF achieves remarkable results in protein structure prediction,^[^ ^1^ ^]^ not all predictions are of equal quality. For many dynamic regions pLDDT scores drop below 70 indicating a less confident or even unreliable prediction of the corresponding atomic positions. Non‐contacting coevolving residues located in dynamic regions may simply arise due to inaccuracies in their positions, or in other cases, these residues may be important for driving the observed dynamics. One such case are residues 29–73 in the periplasmic domain of Tor1I^[^ ^30^ ^]^ (Figure 4c). These residues come up as a hit in our analysis but are located in dynamic loops that have pLDDT scores of ~75 and adopt various conformations experimentally (Figure 4c).

Another class of violations are those that may occur due to residues being functionally rather than physically coupled. Such cases are particularly intriguing as they report on specific residues that are functionally important or may be involved in allosteric regulatory mechanisms. We find one such case in CutA where tryptophan 43 and threonine 78 are coevolutionary coupled as they are involved in ligand binding (Figure 4d). Another example comes from J domain (JD) proteins, a protein family that is of particular interest to our lab.^[^ ^32^, ^33^, ^34^ ^]^ JDs are normally cooperating with Hsp70 in order to maintain cell proteostasis.^[^ ^35^ ^]^ The JD plays an important role in the interaction as it is able to upregulate the ATPase activity of Hsp70 and enhance its function.^[^ ^36^ ^]^ JD uses helices α2, α3 and its HPD motif (Figure 4e) to bind the nucleotide binding domain.^[^ ^37^ ^]^ Together with Dr. Clore, we have recently revealed a previously unknown regulatory mechanism for the JD–Hsp70 interaction that involves helix α5 which blocks Hsp70 binding.^[^ ^32^ ^]^ Application of the presented method to the JD of DNAJB6 (Figure 4e) revealed two pairs of residues that are evolutionary coupled but spatially distant. These involve histidine 32 in the HPD motif which is coupled to arginine 25, and alanine 28 that coevolves with alanine 51. Intriguingly, all of these residues are involved in Hsp70 binding^[^ ^37^, ^38^ ^]^ but also (apart from residue 32) make contacts with α5 (Figure 4e). It is thus interesting to speculate that the observed co‐evolutionary couplings are related with the release of auto‐inhibition and/or binding of JD to Hsp70, a hypothesis that should be tested experimentally.

A final case that needs to be considered is coevolution across hetero‐oligomeric interfaces that ensures effective complex formation, a property often critical for protein function. In line with previous efforts focused on prokaryotes,^[^ ^17^, ^39^ ^]^ the individual MSAs of the two potentially interacting query sequences are combined into a paired MSA (see Methods) that should contain residue correlations across the binding interface. The construction of paired MSAs can be technically challenging and, as not every sequence in the individual MSAs can be paired, can lead to substantial loss in information content. Despite these difficulties, identification of inter‐chain coevolving pairs of residues that do not take part in the interface is particularly interesting as it could indicate conformational changes upon binding or multiple binding modes. Application to the hetero‐dimeric complex of subunits one and two of the molybdopterin converting factor shows that the method is able to correctly predict the hetero‐dimeric interface (Figure 4f). Three out of the 12 predicted coevolution restraints across the interface are not satisfied, an observation that could be attributed to artefacts in the paired MSA but could also indicate alternative binding modes.

## 4 CONCLUSIONS

The evolutionary record of proteins contains rich information about protein structure, and function. We have only recently been able to mine this vast resource using machine learning methods such as AF.^[^ ^26^ ^]^ However, the success of these methods relies mainly in interpreting protein sequences in terms of interatomic contacts,^[^ ^40^ ^]^ while the information regarding protein function, regulatory mechanisms and allostery remains to be exploited. Here a conceptually simple approach was taken towards that direction. Building on the success of AF, a method is presented that identifies coevolving residues that are not in close contact. The main advantages of this approach in comparison with previous efforts^[^ ^25^ ^]^ towards the same goal have to do with: (1) the accuracy of AF models alleviates artefacts related with the atomic resolution of protein structures and (2) the availability of high quality AF models for most of the members of a particular protein family. Even when these issues are taken care of, the vast majority (~65%) of the protein families tested show at least one pair of coevolving residues that are not in contact. A significant portion of these pairs report on the homo‐oligomeric forms of the query sequences,^[^ ^41^ ^]^ and in theory this information could be used to generate small molecules that favour specific oligomeric interfaces.^[^ ^42^ ^]^ Other violations could arise due to protein dynamics or functional regulatory mechanisms as shown in Figure 4. Indeed, distant coevolving residues seem to be significantly more flexible in comparison to their contacting counterparts (Figure 3b) suggesting that they may be involved in conformational changes. Note that the set of sequences tested here is biased towards folded proteins. It will be interesting to see if the method presented can tackle intrinsically disordered regions or proteins (IDRs/IDPs).^[^ ^43^, ^44^ ^]^ In these cases, certain adjustments will be needed as MSAs for these classes of proteins tend to be less deep and direct coupling analysis methods straggle to confidently predict coevolving residues.^[^ ^45^ ^]^ Moreover, AF models of IDRs/IDPs seem to overrepresent the conditionally folded forms of these sequences^[^ ^26^, ^27^ ^]^ an observation that could be taken advantage of in the future. In this study the 20 top scoring hits from CCMpred were selected for analysis as they are normally well above the noise of the prediction, looking deeper in the coevolution matrices (where couplings between flexible residues may be hidden) holds promise to reveal even weaker couplings.

Finally, it is important to stress out that currently, AF structures are not able to effectively capture the conformational space sampled by a protein sequence. However, protein dynamics and allosteric regulatory mechanisms should be captured in the evolutionary record of protein sequences and should therefore be present in MSAs. The ability to generate a set of potentially allosterically coupled residues that are free of false positives (using methods such as the one presented here) is important, but these predictions need to be validated experimentally. Although X‐ray crystallography and cryo‐electron microscopy are excellent tools for probing protein structure, solution NMR methods, of which Dr. Clore is a pioneer,^[^ ^2^, ^3^, ^46^, ^47^ ^]^ are naturally better suited to study protein dynamics and regulatory networks.^[^ ^48^, ^49^ ^]^ It is evident that developing deep learning methods to predict allosteric regulatory mechanisms in combination with solution NMR is an exciting avenue for the field and I hope that the data presented here go a step towards this direction.

## CONFLICT OF INTEREST STATEMENT

The author has not any conflict of interest to report.

## Supporting information


**FIGURE S1:** Analysis of coevolving residues at different pLDDT cutoffs. (a) Distance (left), pLDDT (middle) and CCMpred score (right) distributions for coevolving pairs with an average pLDDT score above 85 that are in contact (blue) not in contact in any of the AF models used in the analysis (red). The same data but for residues with an average pLDDT of below 70 (b) or above 70 (c) are shown. The data have been normalised such that the area under the curve in any case equals to unity. The black dashed line in the left most panels represents 5.0 Å that was used as a contact distance cutoff.

## Data Availability

The data that support the findings of this study are openly available in GitHub at https://github.com/karamanoslab/wmegacouplings.
