# Supplementary material for: Chasing long‐range evolutionary couplings in the AlphaFold era
Source: Biopolymers. 2023 Feb 8;114(3):e23530. doi: 10.1002/bip.23530 (PMC10909459; doi:10.1002/bip.23530)
Supplement: Supplementary file 1 — FIGURE S1: Analysis of coevolving residues at different pLDDT cutoffs. (a) Distance (left), pLDDT (middle) and CCMpred score (right) distributions for coevolving pairs with an average pLDDT score above 85 that are in contact (blue) not in contact in any of the AF models used in the analysis (red). The same data but for residues with an average pLDDT of below 70 (b) or above 70 (c) are shown. The data have been normalised such that the area under the curve in any case equals to unity. The black dashed line in the left most panels represents 5.0 Å that was used as a contact distance cutoff. [file BIP-114-e23530-s001.pdf]

Supplementary Information for

Chasing long-range evolutionary couplings in the AlphaFold era

Theodoros K. Karamanos[1]*

[1]Department of Life Sciences, Faculty of Natural Sciences, Imperial College London

SI Figures

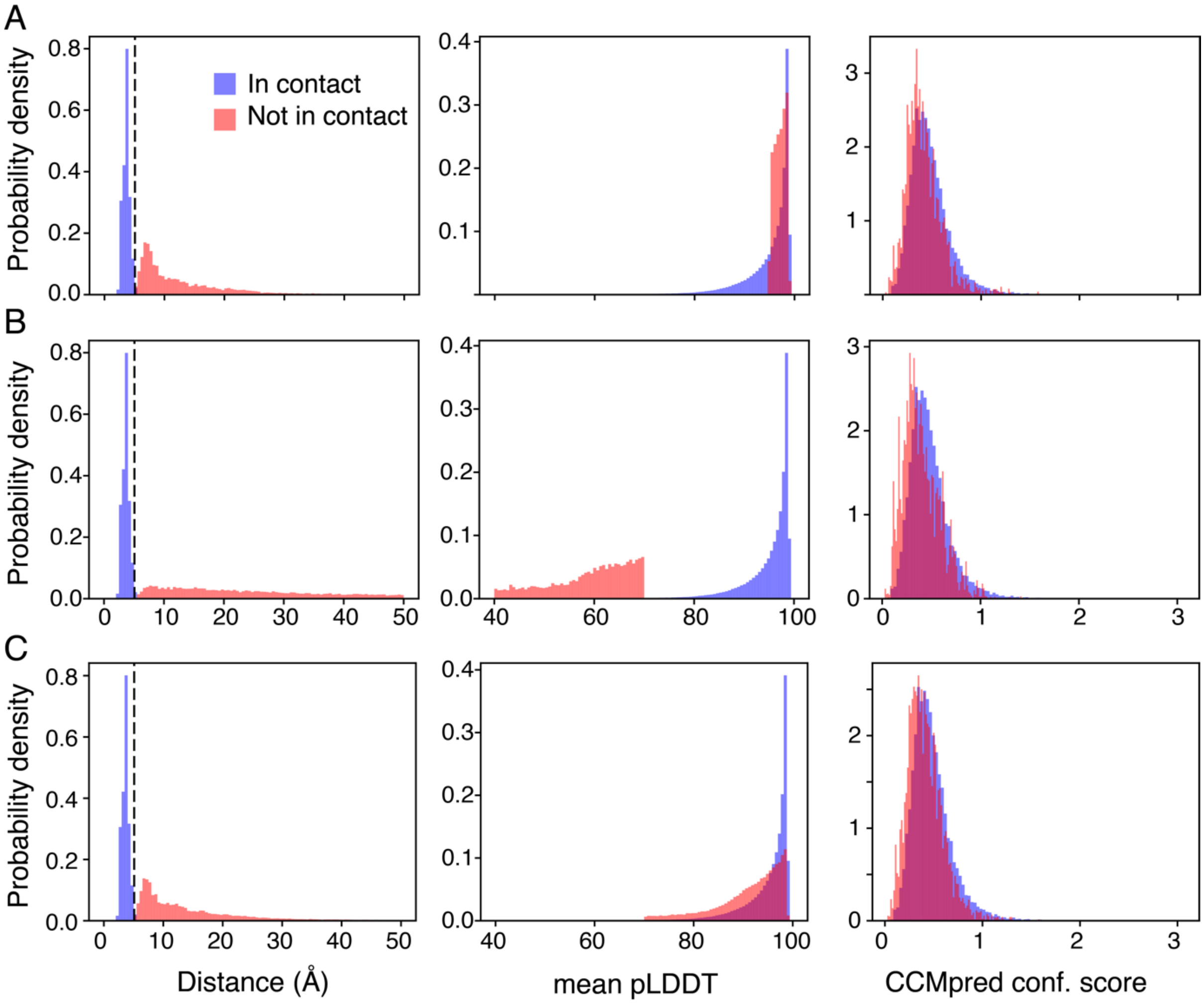

Figure S1: Analysis of coevolving residues at different pLDDT cutoffs. (A) Distance (left), pLDDT (middle) and CCMpred score (right) distributions for coevolving pairs with an average pLDDT score above 85 that are in contact (blue) not in contact in any of the AF models used in the analysis (red). The same data but for residues with an average pLDDT of below 70 (B) or above 70 (C) are shown. The data have been normalised such that the area under the curve in any case equals to unity. The black dashed line in the left most panels represents 5.0 Å that was used as a contact distance cutoff.
